# A Reconfigurable Framework for Vehicle Localization in Urban Areas

**DOI:** 10.3390/s22072595

**Published:** 2022-03-28

**Authors:** Kerman Viana, Asier Zubizarreta, Mikel Diez

**Affiliations:** Faculty of Engineering in Bilbao, University of the Basque Country UPV/EHU, 48013 Bilbao, Spain; kerman.viana@ehu.eus (K.V.); mikel.diez@ehu.eus (M.D.)

**Keywords:** autonomous vehicle, robust localization, reconfiguration, sensor fusion

## Abstract

Accurate localization for autonomous vehicle operations is essential in dense urban areas. In order to ensure safety, positioning algorithms should implement fault detection and fallback strategies. While many strategies stop the vehicle once a failure is detected, in this work a new framework is proposed that includes an improved reconfiguration module to evaluate the failure scenario and offer alternative positioning strategies, allowing continued driving in degraded mode until a critical failure is detected. Furthermore, as many failures in sensors can be temporary, such as GPS signal interruption, the proposed approach allows the return to a non-fault state while resetting the alternative algorithms used in the temporary failure scenario. The proposed localization framework is validated in a series of experiments carried out in a simulation environment. Results demonstrate proper localization for the driving task even in the presence of sensor failure, only stopping the vehicle when a fully degraded state is achieved. Moreover, reconfiguration strategies have proven to consistently reset the accumulated drift of the alternative positioning algorithms, improving the overall performance and bounding the mean error.

## 1. Introduction

### 1.1. Motivation

Accurate localization is a fundamental requirement to achieve a high level of autonomy in navigation and positioning for any autonomous vehicle [1]. To meet this requirement, positioning algorithms render the raw data obtained from the acquisition system, which is composed of different sensors. This rendered output is then used by later systems, such as the trajectory planner, to make decisions based on both the position of the vehicle and its surrounding environment.

In recent years, localization tasks have typically been carried out by fusing global positioning systems (GPS) and inertial navigation systems (INS), mainly due to their affordability and convenience [2]. However, GPS/INS positioning for autonomous vehicles requires ensuring an appropriate-quality GPS signal. This is not always possible in certain areas, such as dense urban areas or zones where signals may be interrupted by tall buildings or tunnels, making vehicle localization unreliable.

For these cases, other localization approaches have been proposed, mainly divided into two groups: (1) local-positioning-based approaches, which use the last known position to estimate relative localization, and (2) map-aided techniques, which offer global localization by using accurate digital maps as global references for the ego vehicle along its driving environment.

One of the earliest approaches for local-positioning-based approaches is the use of dead reckoning techniques, which are typically based on vehicle odometry and INS readings. These techniques have been broadly used in mobile robot localization [3] and, even though accuracy is limited by wheel slippage, terrain inconsistency or vehicle-parameter mismatching, they are still part of the localization strategy of many autonomous vehicles [4,5].

More recently, local positioning has also been addressed by analyzing and processing camera images. This technique has been named visual odometry (VO) [6,7]. A similar approach, which some authors name LiDaR odometry [8], can be implemented using the data cloud provided by LiDAR sensors. In these cases, cloud-point matching or registration algorithms, such as the normal distribution transform (NDT) [9], can be used to estimate the relative orientation and position of the ego vehicle. The use of cameras or LiDAR sensors has the benefit of avoiding drift due to terrain or vehicle parameter mismatches and in recent years have gained increasing interest due to improved accuracy.

However, the aforementioned algorithms, as they calculate relative positions and require integration, will always present drift when used to estimate the position of the vehicle in the global frame. Therefore, these strategies on their own cannot guarantee proper long-term global localization on their own and need to be combined with other approaches.

To cope with the previous limitations, map-aided algorithms have been proposed [10]. These techniques match the approximate localization of the ego vehicle with a predefined mesh of roads, junctions and elements from the driving environment. A popular approach in this area is simultaneous localization and mapping (SLAM), which is focused on localization in unknown scenarios by constructing local maps of the environment while positioning the vehicle in them [11,12]. However, in known urban scenarios, the use of high-definition, globally referenced digital maps can provide accurate global positioning [13]. Two of the main approaches in this field are road matching [14] and feature navigation [15].

Road matching algorithms, based on the premise that the ego vehicle’s position will always be restricted to the road network, aim to integrate previous positioning data with spatial road network data to identify the actual road link on which the vehicle is traveling. Different approaches have been made to solve this issue, and the map-matching algorithms are usually divided into three categories: geometric [16], topological [17] and advanced map matching [14].

On the other hand, feature navigation algorithms [18] consist of a two-step process. First, they need to correctly identify previously mapped and located road elements, such as traffic posts, lights or buildings, surrounding the ego vehicle and captured by the mounted cameras or LiDAR sensors. Often, this step is achieved by the use of classifications algorithms based on artificial neural networks (ANN) [19]. Next, based on the relative distance from the ego vehicle to the identified feature, the position of the ego vehicle is globally corrected from the previous estimation based on relative calculations.

Both of these techniques are broadly used in autonomous driving global localization [15,20]. However, feature navigation algorithms require a two-step process along with a very detailed digital map of the driving environment, which needs to be updated to be accurate. Furthermore, feature navigation works on the assumption that enough features exist in the driving environment to periodically correct the relative localization of the vehicle, which cannot always be guaranteed. On the other hand, road-matching techniques are usually based on simplified maps and are less costly in computational terms. Even in the case of not having a digital map of the driving environment, most of the time this can be easily built using geographic information systems (GIS) [16]. Thus, if the maps are accurate, the latter approach can provide global positioning of the vehicle, making it an interesting complement to GPS-based positioning [10]. Moreover, the previously analyzed local-positioning-based approaches can be combined with map-aided algorithms to correct the accumulated drift [21,22,23].

Therefore, local-positioning-based approaches and traditional GPS/INS approaches may be combined using map-aided techniques to ensure proper localization in dense urban areas. Fusing different sensor data is a widely studied area in the literature [24]. In the case of vehicle localization, the strategies are usually divided into two main groups: optimization techniques and filtering methods. For the former, commonly applied techniques, such as the Bundle Adjustment [25], are used because of their consistent and accurate results on various scenarios. On the other hand, filtering methods tend to be based mainly on extended Kalman filters (EKF) and its variants thanks to their convergence and consistency [2]. Additionally, filters have also offered excellent results by combining different sensors and approaches [26]. Finally, Kalman filtering also offers great versatility regarding state estimation [2], making it the main choice for data fusion in the localization framework.

However, all these approaches are focused on providing accurate positioning assuming that the perception system does not fail or provides incorrect or low-precision data. When considering autonomous vehicles, the localization system not only has to provide accurate positioning but also be able to cope with possible sensor malfunction or signal loss. In this case, defining strategies that can consistently detect possible errors and select the most appropriate sensor data to perform positioning is required so that proper fallback strategies can be executed if needed. This work focuses on this area of research.

### 1.2. Error Detection and Management in Vehicle Localization Systems

The most common strategy for error detection in the sensor inputs of autonomous vehicles is based on duplication and comparison approaches and the use of belief functions, as defined by Smets in his Transferable Belief Model (TMB) [27]; i.e., assembling redundant localization algorithms aiming to compare the different outputs and expose the failing sensor or algorithm. Examples of such approaches can be found in [28,29], where redundant information is used to detect corrupted or erroneous data and obtain control signals that are further fused in a Kalman filter. Self-assessing Bayesian filters have also been proposed to merge different information sources and improve the robustness, as they implement an error detection system [30]. These filters often use of the normalized innovation squared (NIS) metric, which takes measurement information to identify outliers and expose the failure [31]. However, these approaches are based on statistical values that require a certain amount of high-quality measurement and number of samples to ensure the correct detection of failures [32]. Moreover, because of their nature, the use of statistical metrics to detect measurement errors can hardly ensure the instant detection of a failing module, which may lead to wrong decision-making. In general, most of the work in the field of error detection tends to focus only on error-detection consistency, neglecting areas of great interest such as decision-making strategies and alternative-positioning-strategy accuracy.

Once the error has been detected, a decision-making algorithm must determine its effect on the autonomous vehicle. Some authors propose graph-based approaches under the optimization framework [33] and extended Kalman filter (EKF) [30], which can ensure localization in the short term in the presence of errors, even when such errors are not detected. However, these strategies are not valid once the error duration extends for longer periods of time. Therefore, in the presence of failures of longer duration, most of the work in this area proposes to immediately stop the vehicles in a safe place in case of any failure or degradation [34,35].

However, failures in localization modules, particularly in dense urban areas, can be of temporal nature due to signal degradation. This can be due to sensor occlusion, such as the case of cameras or GPS signals, and, in most cases, the signal degradation occurs in particular areas. Hence, immediately stopping the vehicle in these scenarios limits the potential of autonomous vehicles, and some research has proposed implementing fallback strategies based on reconfiguring the positioning algorithms so that the vehicle can continue operating in a degraded mode for a predefined short period of time [36] until either the occlusion or degraded signal is resolved or the error is detected as critical and a stop has to be executed.

In this area, works that propose alternatives are few, and further research is required. Proper fusion of the different localization algorithms to allow overcoming the limitations of each individual sensor in different scenarios, including the possibility of detecting errors while in operation, and reconfiguring the localization framework to compensate for sensor failure or degradation are some of the areas that have not been analyzed in the literature. The present work aims to propose contributions in these areas, as detailed next.

### 1.3. Contributions

In this work, a reconfigurable localization framework is proposed, suitable for dense urban areas and capable of detecting positioning errors and reconfiguring faulty modules accordingly to ensure correct and accurate localization. The proposed framework is an extension of the one presented by the authors in [37] and presents the following main contributions over the works previously analyzed: (1) a localization framework able to cope with different sensor failures thanks to the combination of fusion algorithms and decision strategies; (2) the definition within the framework of a sensor-measurement-based error-detection strategy, which allows determination of which sensor is failing so that the data provided by the failing sensor can be neglected; and (3) a novel reconfiguration module that evaluates the failure scenario and reconfigures the system, adopting alternative localization strategies that use remaining sensor data to avoid vehicle stop until the system is fully degraded.

The proposed reconfigurable localization framework is validated in different simulated urban scenarios against a wide range of possible errors using a CARLA simulator. In this validation, it is to be noted that in this work no new algorithms are proposed for vision, LiDAR or INS/GNSS data processing, as the aim of this work and the validation is not to achieve maximum accuracy. Instead, critical errors are simulated in the sensory system, and validation is focused on the error-detection and reconfiguration strategy proposed in order to demonstrate that the system is able to further enhance operation of the location system by providing the best possible localization.

The remainder of the paper is organized as follows. The localization framework is defined in Section 2, including the error-detection and reconfiguration strategy. Section 3 details the simulation process to test the viability of the localization system in the presence of errors. Finally, the main ideas of this work are summarized in Section 4.

## 2. Reconfigurable Localization Framework

The proposed reconfigurable localization framework is based on a hierarchical localization algorithm structure, an error-detection block and the reconfiguration strategy implemented in a decision block. The hierarchical structure is composed of three levels, each with a different localization approach, ordered from most to least accurate: Level 3 uses algorithms based on calculation of the relative position, using LiDAR, monocular camera, inertial measurement unit (IMU) and odometry; Level 2, based on the previous estimations, adds a digital map to perform road-matching; finally, Level 1 is based on GPS/INS localization. The error-detection block continuously monitors the accuracy of each localization algorithm, providing this information to a decision block that is responsible for defining which level of the hierarchical structure is going to be used. This framework assumes that a low-level lane keeping control and cruise control are implemented in the ego vehicle to assure that the vehicle always remains within the boundaries of the road despite having a failure in the global localization modules.

**Figure 1:**
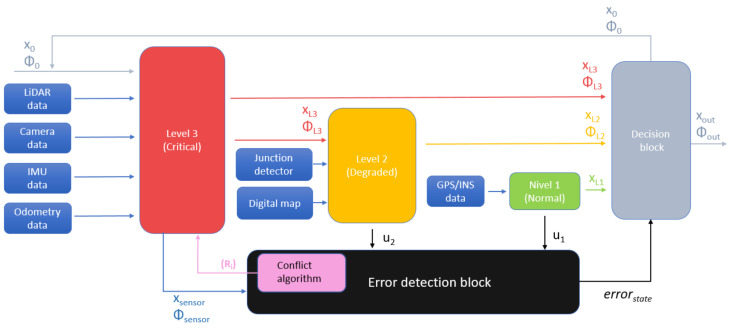
Proposed localization framework.

Figure 1 summarizes the proposed localization framework, including all three hierarchical levels, the output-selection-decision block and the error-detection block. All of these elements will be discussed in depth within the next subsections.

### 2.1. Level 3: Relative Positioning Strategies

This level processes sensor data using approaches focused on relative-localization algorithms. Four different algorithms are implemented in this level, related to each of the four sensor outputs considered: camera, LiDAR, IMU and odometry. For the first, a VO technique is applied based on the one proposed in [12]. For the LiDAR sensor, a LiDAR odometry strategy is applied, based on NDT cloud-point matching or a registration algorithm [38] between consecutive time-steps. Accelerometer and gyroscope readings from the IMU are combined in an extended Kalman filter (EKF) based in measurement integration [39]. Finally, the odometry readings, consisting of the vehicle’s wheel speed and steering angle, are used to feed a bicycle model for the ego vehicle [40] and estimate the location of the vehicle by integration [5].

The previous approaches are based on computing the relative displacement and vehicle’s yaw-angle variation between consecutive time-steps. Hence, to estimate the localization of the ego vehicle $x_{i}$, an initial known position and orientation from the previous time step must be provided, $x_{i-1}$ and $\phi_{i-1}$, respectively:

(1) $$\left[\begin{array}{c} x_{i}\\ y_{i} \end{array}\right]=d\cdot \left[\begin{array}{c} \cos(\phi_{i})\\ \sin(\phi_{i}) \end{array}\right]+\left[\begin{array}{c} x_{i-1}\\ y_{i-1} \end{array}\right]$$

(2) $$\begin{array}{c} \phi_{i}=\Delta \phi +\phi_{i-1} \end{array}$$

where *d* defines the estimated distance variation between two steps.

In order to provide a more robust output, the four algorithms previously defined are fused using an extended Kalman filter, which estimates the ego vehicle position $x_{L3}$ and orientation values $\phi_{L3}$. These two values will be propagated to the next level, becoming the starting estimation of the map-matching algorithm.

As previously detailed, algorithms based on relative positioning present drift over time due to the accumulation of small errors in the calculated relative displacements. Therefore, if the decision block selects only this level as the output of the localization framework, the system will be considered in a critical state, as it is not suitable for long-term accurate navigation.

### 2.2. Level 2: Road-Matching Algorithms

This level combines the estimation provided by Level 3 with a geometry-based, point-to-point, road-matching algorithm [10], providing more accurate global localization estimations $x_{L2}$ and $\phi_{L2}$. Besides that, an HD map, given by CARLA, is used as an extra input or sensor for the localization task, similar to the most common road-matching algorithms. However, road-matching algorithms present two main issues that compromise their accuracy: mismatches in junctions, mainly where many roads come together, and longitudinal drift, which may accumulate over time despite correctly matching the road link. These errors may lead to an incorrect link selection by the road-matching algorithm.

To deal with these issues, the proposed work simulates a simple feature-based correction algorithm, similar to [13], to detect road junctions and match them with their position on the digital map. This way, every time the vehicle reaches a junction, the previous position estimation can be corrected based on the global position of the detected junction. It should be underlined that this method assumes that the junction-detection algorithm always works correctly, based on the premise that an autonomous vehicle cannot follow its trajectory and make decisions accordingly without properly functioning junction detection.

Level 2 localization can provide appropriate global position and orientation information for a limited time, as the proposed approach will present longitudinal drift, the correction of which will depend on the detection of the aforementioned feature (junctions). Therefore, a localization system working at Level 2 will be considered degraded.

### 2.3. Level 1: GPS/INS

Level 1 implements the most accurate global localization approach on this framework, based on the widely used GPS/INS localization approach that combines GPS data with INS sensor data using an integrated Kalman filter [2]. It is assumed that this signal requires no further computation to estimate vehicle position, $x_{L1}$. This localization technique is the most accurate one since it is not susceptible to external factors such as vehicle parameter mismatches or accumulated drift, although this does not always ensure absolute accuracy. Moreover, its only error source comes from its own sensor-reading precision and the external signal it receives from satellites.

Therefore, assuming that in an ideal situation the readings of the GPS/INS sensor will provide the most accurate estimation, this level will always have the top priority in the localization structure. Hence, a localization system working at Level 1 will be considered as normal behavior, as it is suitable for fully accurate long-term navigation.

### 2.4. Error-Detection Block

The error-detection block is responsible for evaluating the estimation provided by the aforementioned three levels and determining if there is a failure in one of the levels by modifying the $error_{state}$ variable, which will later be processed by the decision block to reconfigure the output of the localization system.

The error-detection block has been designed to consider the following failures and malfunctions:

- Level 1: the GPS/INS-based localization level’s accuracy is mainly affected by the satellite signal. Therefore, its malfunction will always come from obstruction (quality degradation) or lack of this signal.
- Level 2: similar to the Level 1 error source, map-matching algorithm accuracy is strongly linked to the digital map precision and availability. Therefore, in case the vehicle enters an area where there is no map data or it is not accurate, the map-matching algorithm will be tagged as inaccurate.
- Level 3: sensors may become faulty because of wear, failure or a change in the environment that affects the nature of their readings. Some examples of possible environmental changes are: low visibility during night or twilight for cameras, presence of lightning or strong flashes for the LiDAR or tire slippage for vehicle odometry and INS readings.

In order to provide a robust localization approach in the error-detection block, strategies to detect the aforementioned effects are implemented. For that purpose, two main approaches are followed, depending on the hierarchical level analyzed. For Levels 1 and 2, two control signals have been implemented to evaluate GPS signal quality, $u_{1}$, or map precision, $u_{2}$. Note that GPS devices typically provide this information based on satellite signal strength and the number of detected satellites. In the case of digital maps, error can be easily detected by the vehicle moving into an uncharted area not defined on the map.

**Figure 2:**
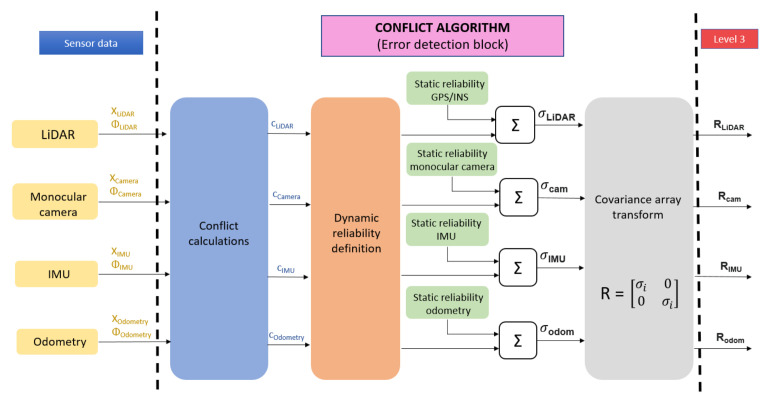
Conflict algorithm structure.

On the other hand, the algorithms integrated into Level 3 directly use each sensor estimation data, both for position and orientation. Hence, for this level, a conflict evaluation approach has been developed to detect failure of one of the sensors (see Figure 2). The proposed algorithm is based on the one proposed in [41] and uses a duplication-comparison technique that computes a dynamic reliability value for each source. The proposed approach can detect faulty sensors by comparing the conflict or discrepancy between the output estimations provided by each sensor (and its corresponding processing algorithms, as detailed in Section 2.1) using the following equation:

(3) $$c_{sensor_{i}}=\frac{1}{\left(n-1\right)}*\sum_{k=1}^{n-1}\left[\begin{array}{c} x_{i}\\ \phi_{i} \end{array}\right]-\left[\begin{array}{c} x_{k}\\ \phi_{k} \end{array}\right]$$

where $x_{i}$ and $\phi_{i}$ represent the position and orientation estimation for the sensor whose conflict is being evaluated, prior to the Level 3 EKF filter fusion; $x_{k}$ and $\phi_{k}$ are the position and orientation for the rest of the sensors; *n* is the number of sensors integrated in the framework (four in this approach) and $c_{sensor_{i}}$ is the conflict value associated with each sensor.

After calculating the conflict value $c_{sensor_{i}}$, it is compared with a threshold value $\bar{c_{sensor_{i}}}$, which is set experimentally as 2.5 m for the position estimations and 5 degrees for the orientation estimation. If this value is surpassed, a dynamic reliability value $\sigma_{din_{i}}$ is calculated as follows:

(4) $$\left\{\begin{array}{ccccc} \sigma_{din_{i}} & = & 0 & \; & c_{sensor_{i}}\leq \bar{c_{sensor}}\\ \sigma_{din_{i}} & = & c_{sensor_{i}}+f\cdot e_{i} & \; & c_{sensor_{i}}>\bar{c_{sensor}} \end{array}\right.$$

which defines the dynamic reliability value proportion *f* to the maximum deviation of the evaluated estimation from the rest of the estimations $e_{i}$. Variable *f* is set empirically to 2.5 for the orientation estimation and 1 for the position estimation. The dynamic reliability value is added to the static reliability of each source $\sigma_{est_{i}}$, which characterizes the expected accuracy for each sensor–algorithm pair and is based on each algorithm’s expected accuracy as found in the literature, allowing obtainment of the global reliability $\sigma_{i}$:

(5) $$\sigma_{i}=\sigma_{din_{i}}+\sigma_{est_{i}.}$$

Finally, this last value is transformed into the measurement noise covariance matrix $R_{i}$ as follows:

(6) $$R_{i}=\left[\begin{array}{cc} \sigma_{i} & 0\\ 0 & \sigma_{i} \end{array}\right].$$

This value is introduced into the EKF used to fuse the different outputs in Level 3. This way, whenever one of the sources becomes faulty, its dynamic reliability value will grow and further propagate to the covariance matrix, making the EKF lower the weight of this same source in the final output, eliminating or mitigating the error influence. The main advantage of this approach is the instant detection of malfunctioning systems, as its detection is based on immediate measurements and not statistical metrics.

Figure 2 depicts the structure of the implemented conflict algorithm for Level 3, along with the global reliability transformation into an EKF measurement noise covariance matrix for each source.

Note that the proposed conflict algorithm needs a minimum of three different sources to detect a single faulty source [42]. Thus, there is a limit on the number of sensor failures that the conflict evaluation system will be able to detect. Whenever this limit is exceeded, conflict between all sensors, both the failing and correctly working ones, grows above the threshold, and the system cannot be trusted anymore.

### 2.5. Decision Block

This decision or reconfiguration block receives the estimations calculated in the three Levels and the error state of each level, characterized by the variable $error_{state}$, and defines the output of the localization framework and its state (normal, degraded, critical or emergency). The behavior of this block is summarized in Table 1. Note that this table illustrates the failures that require reconfiguration of the localization framework’s output.

It is to be noted that, as previously detailed, reconfiguring the framework’s output to the estimation provided by Level 2 or Level 3 does not ensure long-term accuracy. Hence, time thresholds are included for the degraded and critical cases. For the first, the time threshold defines the maximum time the Level 2 algorithm can safely operate without resetting the longitudinal drift using the junction-detection algorithms. A watchdog timer is initialized when entering the degraded state and resets each time the longitudinal drift is corrected. However, if this correction is not possible, an emergency stop signal will be generated (marked with *). Besides that, a correction distance threshold is also implemented in Level 2. Once a junction is detected (based on feature detection), the closest junction position on the digital map will be searched. An emergency stop signal will also be generated, if no junction is found within a confidence distance value.

For the case of Level 3, even if the internal EKF ensures the best possible localization accuracy, a time threshold is defined in which the vehicle should perform a fallback strategy. If the time is reached, a emergency stop signal will be generated. Furthermore, if the Level 3 localization system ever accumulates too many faulty sensors to be trusted, the decision block will send an emergency signal to find a safe stop for the vehicle.

One of the main contributions of this work is the concept of reconfiguration between the different Levels, which allows handling of short failures, such signal loss or intermittent failures, allowing continued operation of the vehicle. A clear example of these failures are GPS signal losses when driving through dense urban areas, as satellite signals are lost and recovered constantly. In these cases, the proposed approach takes advantage of the accuracy of higher levels to include a drift reset for lower ones.

## 3. Validation

In this section the proposed localization framework will be tested and validated in a realistic simulation environment in which different failure scenarios will be evaluated.

### 3.1. Simulation Setup

In order to validate the proposed approach, a set of simulated scenarios have been implemented in the CARLA Simulator environment, particularly in the custom scenario “Town 03” [43]. A Tesla 3 vehicle has been selected as the study case, equipped with GPS/INS, LiDAR, monocular camera, IMU and wheel speed and steering angle odometry sensors. Data from CARLA is transferred using the Python API to Matlab/Simulink, where the proposed localization approach has been implemented. Regarding the emergency signal control within the decision block, for Level 2 a 30 s time threshold and a 10 m distance threshold have been implemented, and for Level 3, a 15 s threshold.

Twelve different scenarios are proposed to validate the localization framework. All scenarios vary in their routes, but they share the same error pattern, listed in Table 2. As can be seen from the table, Level 1 and 2 failures are intermittent with varying recovery times. On the other hand, Level 3 sensor failures are not recovered: after 1 min the LiDAR measurements start to fail and after 2 min odometry will become unreliable, which will put the localization system just within the limit of its functionality during the last critical state. All these failures are simulated by manually disrupting the CARLA sensor modules once the readings have been received. The disruption magnitudes are simulated according to the literature and the recorded errors for each sensor [2,12,44]. This way, the setup aims to test the proposed approach in the presence of a set of longer errors—situations in which graph-based optimization or other EKF strategies fail. The main objective of this simulation is to validate the robustness of the localization framework, the conflict-based error-detection strategy and the drift-reset strategy for lower levels.

### 3.2. Simulation Results

Table 3 summarizes the simulation results for scenarios that complete their trajectories, i.e., scenarios where the decision block does not send an emergency signal to stop the vehicle. To evaluate these results a mean-error-based criteria is applied, based on the results of similar works in this area [31,32]. Still, it should be underlined that the referenced works’ approaches do not consider sensor failures in their results. Therefore, based on these works, a mean error higher than 3 m in the longitudinal and 1 m in the lateral positioning are set as criteria to determine the scenario of a malfunction. This is also the reason for not including additional statistical measures of the performance, such as maximum error values or CDF graphs. The presented framework is focused on guaranteeing that in the presence of degraded situations localization can be estimated until a critical failure arises, and that the system is able to reconfigure itself and correct the position estimation despite the appearance of peak error values.

Note that the results shown in this section will be analyzed considering longitudinal and lateral errors independently, as the proposed framework’s performance differs significantly for each error. For instance, longitudinal errors are prone to increment due to the drift generated from map-matching algorithms from Level 2, as they lack a strategy to globally position in the longitudinal axis unless a feature-navigation strategy is implemented. This effect is seen in all simulations detailed in Table 3, where the mean longitudinal error is higher than the lateral error. In order to cope with this issue, a junction-detection algorithm is implemented in this work, which bounds the longitudinal accumulated drift whenever a junction is detected. From the global results related to these scenarios, we can derive two main conclusions: (1) the proposed localization framework is suitable for long-term positioning, even in the presence of failures, without the need of a vehicle stop, and (2) the reconfiguration strategies consistently reset the accumulated drift of less-accurate localization algorithms, working in the presence of failing scenarios to improve overall accuracy.

**Figure 3:**
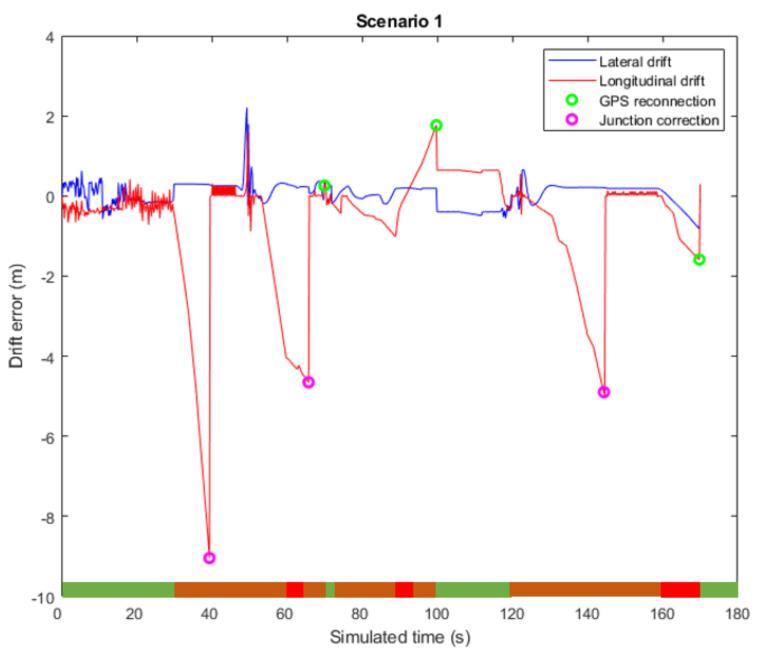
Lateral and longitudinal deviation for Scenario 1. Different working modes of the framework along the test are depicted in an horizontal colored bar. The green colored bars, refer to the NORMAL BEHAVIOR state; the orange colored bars, to the DEGRADED state; and, finally, the red colored bars, to the CRITICAL state.

**Figure 4:**
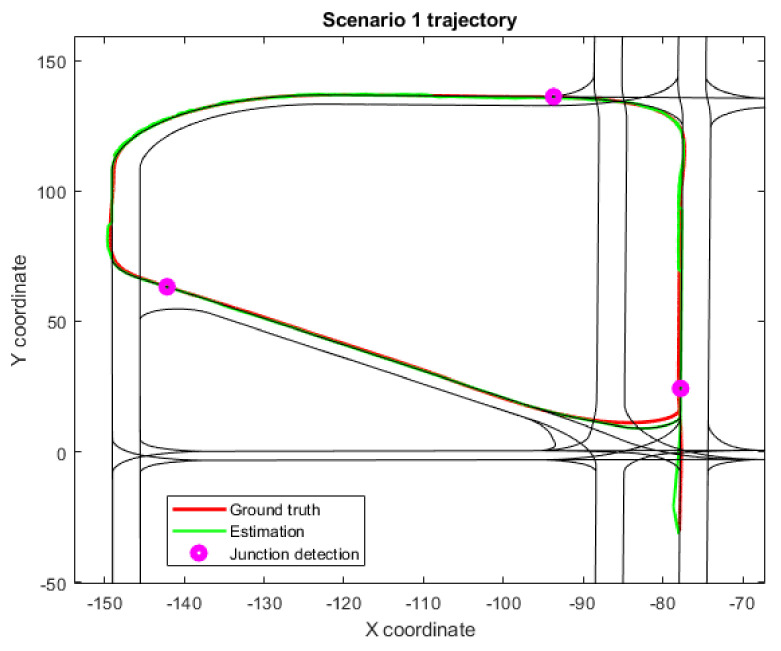
Trajectory for Scenario 1, which shows a properly working scenario results; including the estimated position, the real or ground truth position and the location where the junction-detection algorithm activates.

As an example of a properly working scenario, Figure 3 summarizes the lateral and longitudinal drift along the trajectory of Scenario 1, which is depicted in 2D in Figure 4. The former shows different working modes of the framework along the test depicted in a horizontal, colored bar. The green bars refer to the NORMAL BEHAVIOR state, with Level 1 localization as the framework output; the orange bars are the DEGRADED state, in which Level 2 road-matching localization is being used; finally, the red bars are the CRITICAL state, where Level 3 localization acts as the only remaining reliable output. Besides that, the junction-detection algorithm is activated several times during Scenario 1, as is marked in Figure 3 and Figure 4. In this scenario, the effect of longitudinal drift is clearly seen in the first DEGRADED state (seconds 30 to 60 as defined in Table 2). In this case, Level 2 positioning is working, and there is no global reference to correct the longitudinal drift until a junction is detected (purple circle), which allows drift correct. Lateral error, however, remains bounded in most cases, as additional sensors are able to correctly position the vehicle within the road limits.

**Figure 5:**
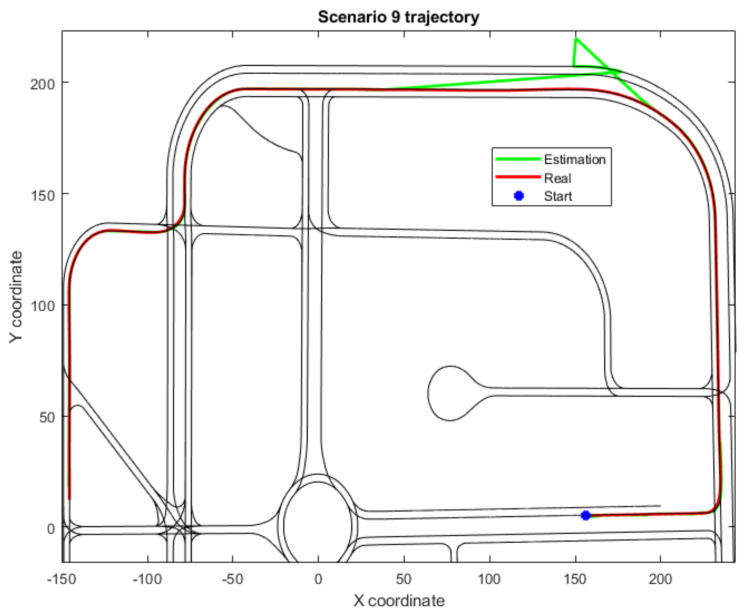
Trajectory for Scenario 9, where the system has reached a malfunctioning state; also includes estimated position and real or ground-truth position.

On the other hand, Figure 5 depicts the trajectory of Scenario 9, exposing the reason for its malfunctioning. Due to a combination of a critical error along with a sharp turn in the trajectory, the localization framework drifts the vehicle’s localization too far from its real position—far enough to make the reconnection with the Level 2 algorithms inconsistent within the map’s road network and the vehicle’s trajectory. Furthermore, as no junctions are detected during this period and the duration of the threshold is not surpassed, emergency signals cannot be sent to stop the vehicle. Therefore, until the Level 1 module is available again, the localization is incapable of either resetting the drift or positioning the vehicle properly. Once this reconnection is achieved, the localization framework correctly positions the vehicle for the rest of the trajectory.

**Figure 6:**
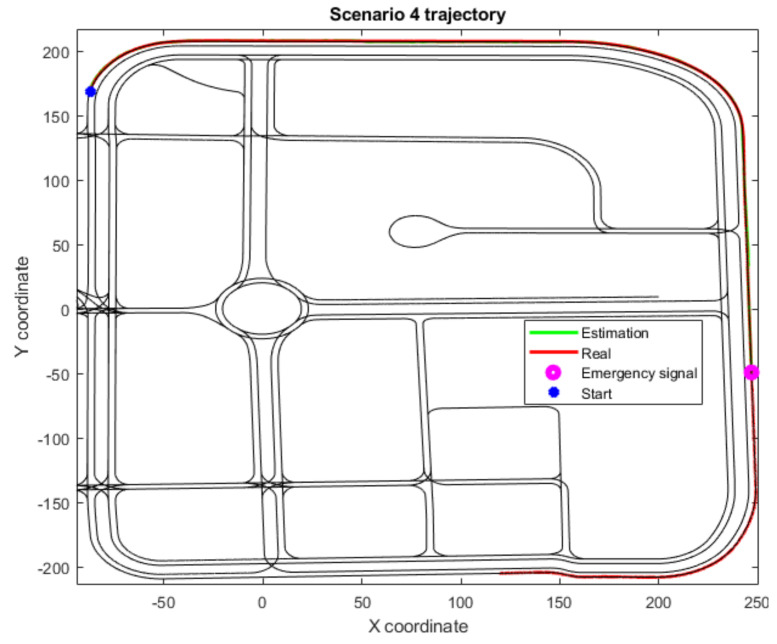
Trajectory for Scenario 4, with an emergency stop because of the Level 2 time threshold; also includes the estimated position, the real or ground truth position and the location where the junction-detection algorithm activates.

**Figure 7:**
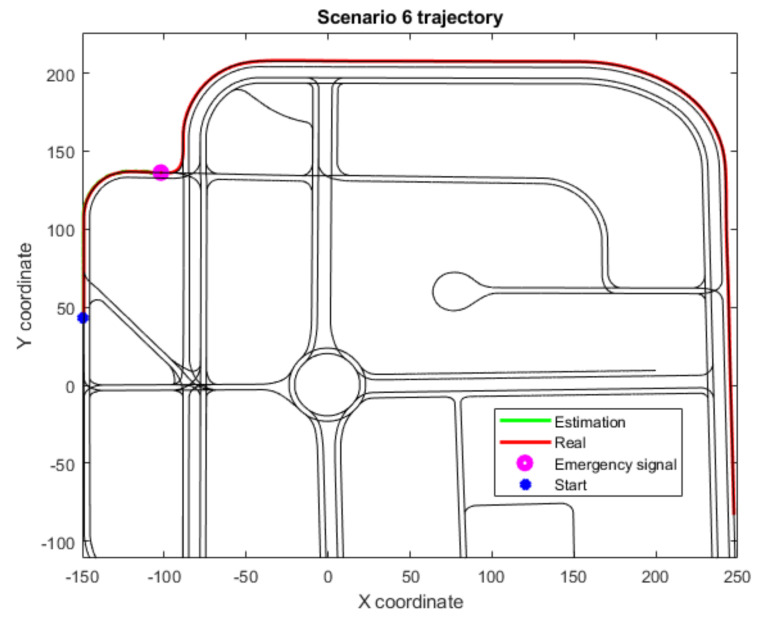
Trajectory for Scenario 6, with an emergency stop because of the Level 2 time threshold; also includes the estimated position, the real or ground truth position and the location where the junction-detection algorithm activates.

**Figure 8:**
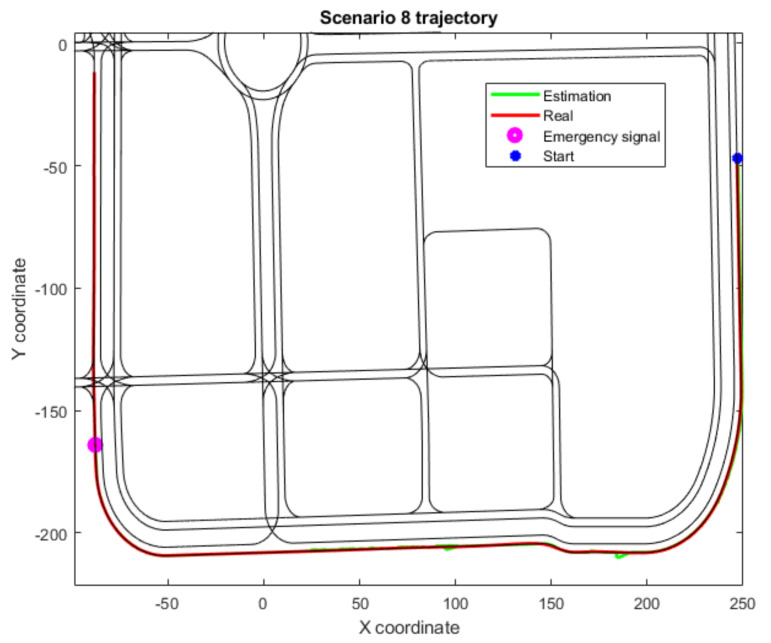
Trajectory for Scenario 8, with an emergency stop because of the Level 2 correction distance threshold; also includes the estimated position, the real or ground truth position and the location where the junction-detection algorithm activates.

In addition, three scenarios (4, 6 and 8) have not been included in Table 3 as they have not completed their trajectories due to an emergency stop signal. All three of these scenarios include fewer junctions, and, therefore, the deviation in these cases increases as longitudinal drift corrections cannot be made often enough. However, thanks to the Level 2 threshold limit implementation, the vehicle is stopped before the drift grows unbounded, as at that point the degraded state cannot be trusted. This effect is shown in the trajectories for Scenarios 4 and 6 (Figure 6 and Figure 7), where the localization framework has been working continuously with the Level 2 algorithms for a time that surpasses the defined threshold. In Scenario 8 (Figure 8), a junction is detected but cannot be matched with any junction within the threshold distance. Hence, an emergency signal is sent to stop the vehicle, as it considers the global localization presents high estimation errors.

**Figure 9:**
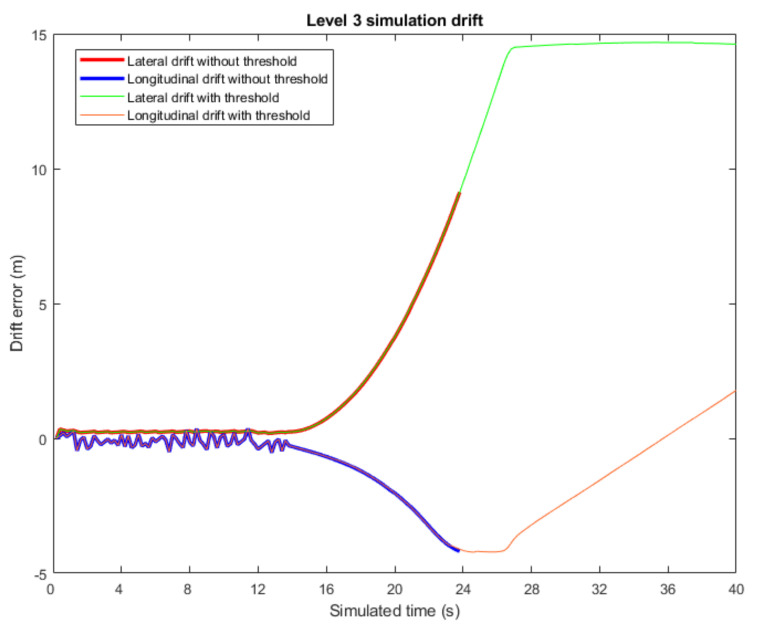
Drift values in longitudinal and lateral axis for both simulations in Level 3 test.

**Figure 10:**
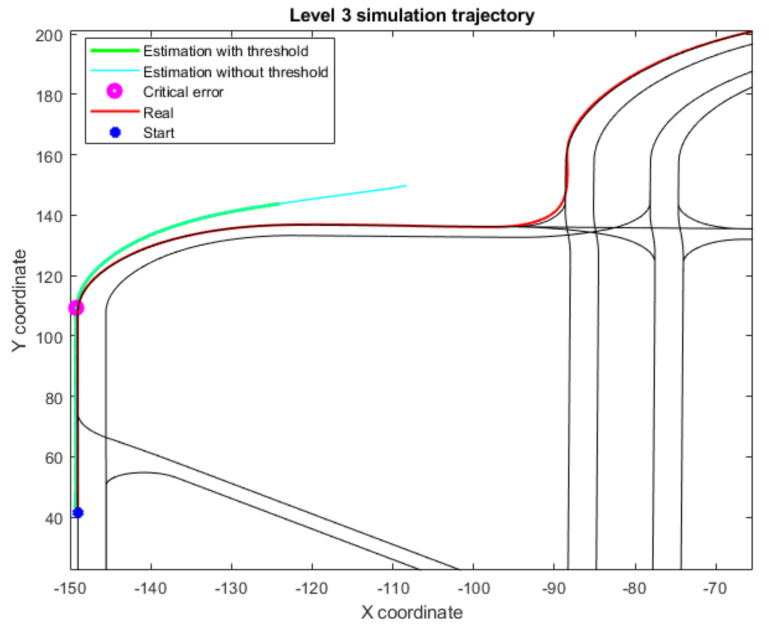
Trajectory for both simulations in Level 3 test; includes the regular threshold implementation and its lack.

Finally, to prove the need of the Level 3 time threshold, another test has been carried out, studying the response of the proposed approach both with a time threshold and without it. In both simulations, the same error pattern has been implemented: a critical error at time-step 14 s lasting until time-step 40 s. As depicted in Figure 9, when there is no threshold to stop the vehicle, the error metrics keep growing unbounded. Moreover, as the error is not bounded, the reconnection with Level 2 algorithms is not viable, as seen in the trajectory pattern defined in Figure 10. Therefore, the implementation of a time threshold for Level 3 not only limits the error metrics but also assures successful reconnection once the map is available again.

## 4. Conclusions

This paper proposes a reconfigurable localization framework for dense urban areas, where sensor or signal failures are frequent and usually compromise vehicle positioning. The approach is based on a hierarchical structure based on GPS/INS, road-matching and relative localization calculations and differentiated by levels along with an error detection module to evaluate the state of each level and a decision block in charge of reconfiguring the system in the presence of failures. The validation, based on simulated scenarios, proves the reconfiguration capabilities of the framework in the presence of a wide range of errors and concurrently shows that the implemented reconfiguration strategies allow the selection of the most accurate sensor data to perform the localization, enhancing the operational limits of the localization system until the errors are critical.

## Figures and Tables

**Table 1:** Decision block strategies. The asterisk indicates that an emergency stop signal is generated.

Failures	Local. Output	State	Emerg. Stop?
None	Level 1	Normal behaviour	No
Level 1	Level 2	Degraded	Yes *
Level 1 Level 2	Level 3	Critical	Yes *
Level 1 Level 2 More than 2 faulty sensors at Level 3	None	Emergency	Yes

**Table 2:** Error pattern for 12 scenarios.

Simulation Time (s)	Failure	Recovery Time (s)	Duration (s)
30	Level 1 lost	70	40
60	Level 2 lost	66	6
60	IMU lost	-	-
72	Level 1 lost	100	28
90	Level 2 lost	96	6
106	Level 2 lost	112	6
120	LiDAR lost	-	-
120	Level 1 lost	170	50
160	Level 2 lost	172	12

**Table 3:** Error metrics for test scenarios with full trajectory.

Test Scenario	Length (m)	Lateral Mean Error (m)	Longitudinal Mean Error (m)	Combined Mean Error (m)
1	629.51	0.24	0.97	3.35
2	700.85	0.17	2.31	3.9
3	691.17	0.26	0.86	7.38
5	626.15	0.32	2.34	5.74
7	627.82	0.21	1.46	4.79
9	766.42	0.49	3.49	8.29
10	597.21	0.53	1.75	2.86
11	524.37	0.27	1.47	4.09
12	588.57	0.26	2.46	2.86
